# A case report of Mikulicz's disease

**DOI:** 10.11604/pamj.2020.37.252.26835

**Published:** 2020-11-19

**Authors:** Dang Luu Vu, Tra My Thieu-Thi, Minh Thong Pham, My Le-Thi, Huu An Nguyen, Minh Duc Nguyen

**Affiliations:** 1 Radiology Center, Bach Mai Hospital, Ha Noi, Vietnam,; 2 Department of Radiology, Ha Noi Medical University, Ha Noi, Vietnam,; 3 Department of Radiology, Pham Ngoc Thach University of Medicine, Ho Chi Minh City, Vietnam,; 4 Department of Radiology, Children's Hospital 2, Ho Chi Minh City, Vietnam

**Keywords:** Mikulicz’s disease, lacrimal gland, salivary gland, swelling, case report

## Abstract

Mikulicz's disease is a unique condition involving the enlargement of the lacrimal and salivary glands, similar to that observed in Sjogren's syndrome; however, Mikulicz's disease is clinically characterized by infrequent autoimmune reactions and responsiveness to glucocorticoid treatment. The ultrasound features of the lacrimal and salivary glands in patients with IgG4-Mikulicz's disease were characterized by multiple hypoechoic areas of varying sizes within the enlarged glands. IgG4 serum level was also elevated, in contrast to the detection of normal levels in Sjogren's syndrome. In this article, we intended to illustrate a case of Mikulicz's disease with clinical and imaging features.

## Introduction

IgG4-related disease (IGG4-RD) is a chronic inflammatory disease, characterized by swollen and thickened lesions of the affected organs, such as lacrimal glands, salivary glands, or pancreas, with high serum concentrations of IgG4, and marked IgG4-positive plasma cell infiltrations of the affected tissues. Inflammation can extend to multiple organs, including the biliary tree and kidney, during long-term follow-up. Therefore, IgG4-RD is generally regarded as a systemic disease. Diseases involving lacrimal and major salivary glands have often been referred to as “Mikulicz´s disease” or “Mikulicz´s syndrome” in the past [1]. Since the first case was reported by Johann Mikulicz, in 1892, Mikulicz´s disease (MD) has remained a controversial topic, with a variety of proposed etiologies. Mikulicz described this disease as a non-malignant secondary chronic inflammation [2]. Schaffer and Jacobson suggested that MD should be reserved for idiopathic cases that follow a benign course, whereas Mikulicz syndrome should be used to describe cases associated with a known underlying disorder [3]. In 1933, Sjogren designated MD as a subtype of Sjogren's Syndrome due to the similar histological features of these two entities [4]. However, several discrepancies also exist, with the minimal detection of keratoconjunctivitis sicca and xerostomia and the absence of Sjogren-specific anti-SSA and anti-SSB antibodies in MD [5]. The infiltration of IgG4 plasma cells into the lacrimal, parotid, submandibular glands and other organs and the response to treatment can also differentiate MD from Sjogren's syndrome [6]. In this article, we aimed to illustrate a case of Mikulicz´s disease.

## Patient and observation

A 32-year-old female patient was admitted to hospital with bilateral, symmetrical, painless swelling of the lacrimal, submandibular glands, with no history of dry eyes or mouth. The symptoms appeared since the first pregnancy and lasted for a period of longer than 3 years. Although she attended medical check-ups several times, she did not receive any definitive diagnosis or treatment.

**Figure 1:**
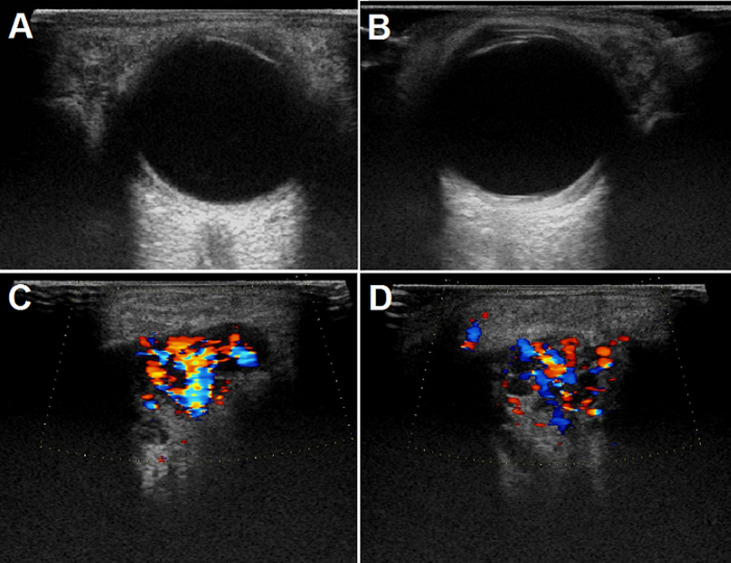
ultrasound of the lacrimal glands before treatment: the left (A) and right (B) lacrimal glands were enlarged (left: 30 x 14 mm and right: 26 x 14 mm) before treatment, with multiple hypoechoic areas; C, D) both glands displayed hypervascularity

**Figure 2:**
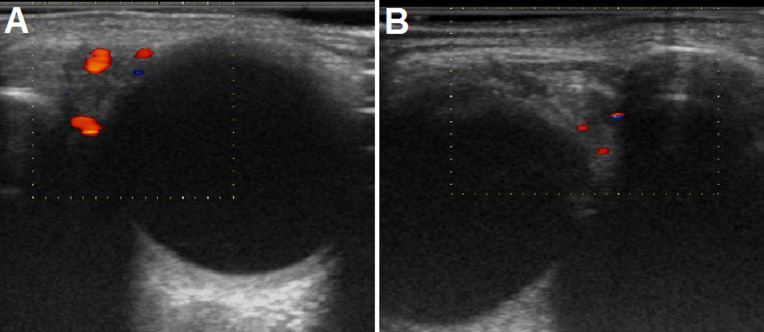
ultrasound of the lacrimal glands after treatment: left (A) and right (B) lacrimal glands decreased in size (left: 9 x 4 mm and right: 8 x 5 mm), and the vascularity and hypoechoic areas disappeared in response to corticosteroid therapy

**Figure 3:**
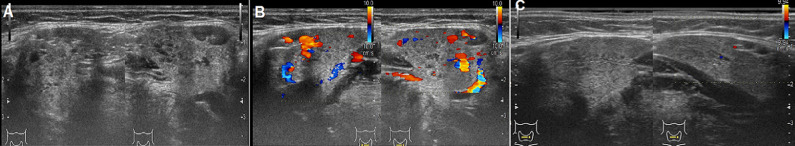
ultrasound of the submandibular glands: A, B) the submandibular glands before treatment showed bilateral enlargement with multiple hypoechoic, hypervascular areas; C) the submandibular glands after treatment showed a decrease in the size and vascularity of the glands, in addition to the disappearance of hypoechoic areas

**Figure 4:**
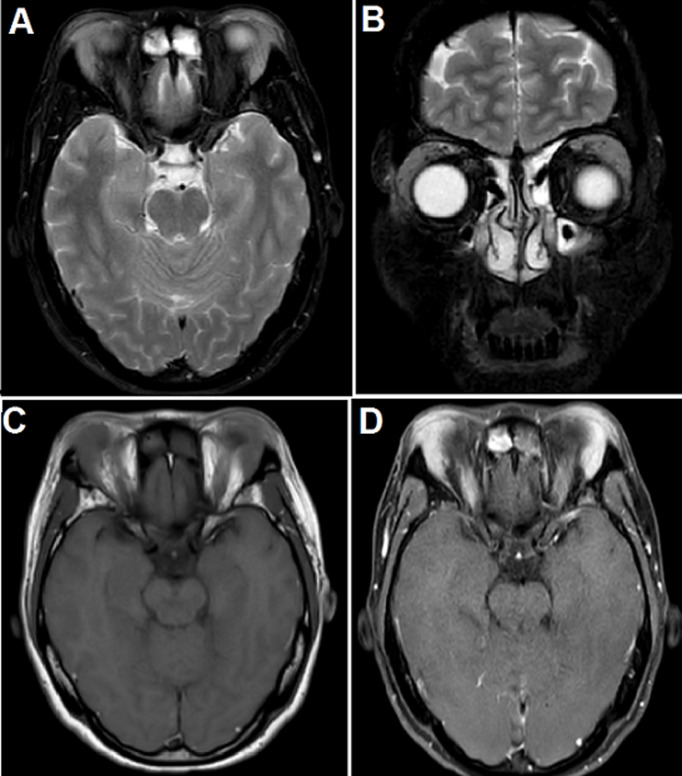
MRI showed enlarged lacrimal glands before treatment: A, B) fat-suppressed T2-weighted images demonstrated the hypointense bilateral swelling of the lacrimal glands; C) the lacrimal glands were isointense to skeletal muscle on T1-weighted images; D) T1-weighted images post-contrast showed that the lacrimal glands are remarkably and homogeneously enhanced

**Figure 5:**
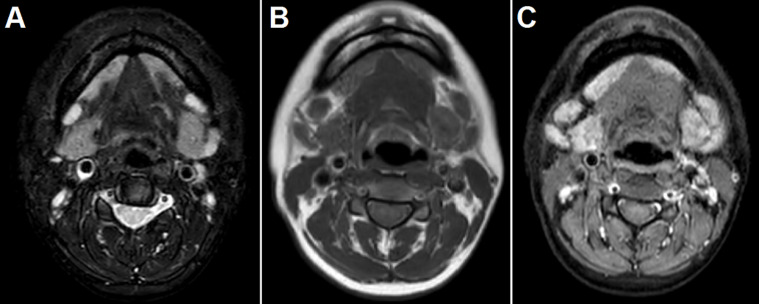
facial MRI before treatment: A) fat-suppressed T2-weighted images showed that the submandibular glands were diffusely enlarged and hypointense; B) T1-weighted images demonstrated the low signal intensity of submandibular glands; C) T1-weighted images with contrast showed the remarkably homogeneous enhancement of these glands

**Imaging findings:** on ultrasound examination of the patient´s orbit and neck, the diffuse enlargement of the lacrimal and submandibular glands was observed, in addition to hypervascularity and multiple hypoechoic areas (Figure 1, Figure 2, Figure 3). Facial magnetic resonance imaging (MRI) demonstrated the diffuse enlargement of the lacrimal and submandibular glands, without evidence of focal lesion or nodularity. The glands were hypointense on T2 signal with marked enhancement. No inflammatory stranding or infiltration was observed surrounding the glands. No evidence of sialolithiasis or dilatation of the parotid duct was detected (Figure 4, Figure 5).

**Figure 6:**
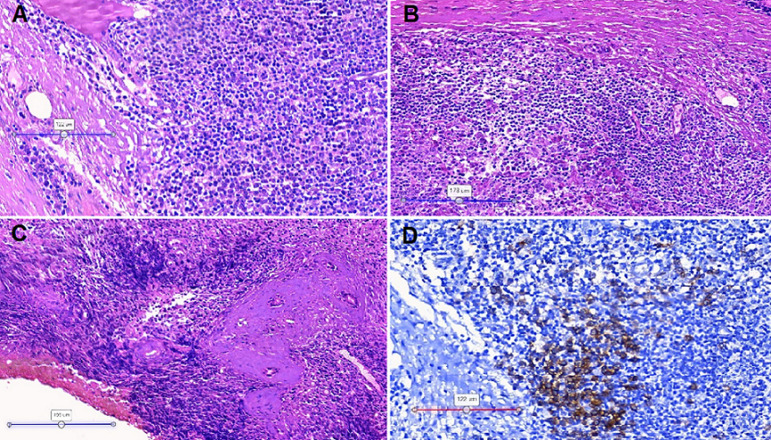
microscopic images of the lacrimal gland visualized with hematoxylin and eosin stain (A-C) and CD138 antibody stain (D); A) lymphoplasmacytic infiltration and fibrosis; B) fibrosclerosis was found in the gland interstitium; C) lacrimal ducts were observed with collagenous sheaths; D) CD38 immunoreactivity was observed, confirming plasma cell infiltration

**Figure 7:**
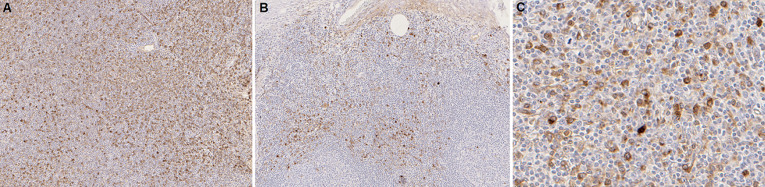
microscopic images of lacrimal gland with anti-IgG antibody stain x100 (A); anti-IgG4 antibody stain x100 (B) and anti-IgG4 antibody stain x400 (C); A, B) representative section of the lacrimal gland showed infiltration of IgG and IgG4 cells; C) markedly increased IgG4-positive plasma cells (the number of IgG4+ cells >50/ high-power field)

**Pathology:** the patient underwent a biopsy of the lacrimal gland. The microscopic images showed lymphoplasmacytic infiltration and fibrotic areas. The immunohistochemical findings revealed CD138-positive cells (Figure 6) and the absence of malignant cells. Immunostaining results demonstrated that there were remarkably increases of IgG and IgG4 positive plasma cells with the IgG4 positive plasma cells > 50/ high-power field (Figure 7).

**Serum IgG4:** serum IgG4 levels were as high as 5,012 mg/L before the administration of steroid therapy, compared with the normal range of less than 1,350 mg/L [7].

**Treatment:** the patient was treated with 40 mg/day prednisolone for 4 weeks, after which the dose decreased by 5 mg/week until it reached 5 mg/day. After 9 months of treatment with corticoids, the serum IgG4 level was markedly decreased, returning to within the normal range, at 798 mg/L, accompanied by decreased gland sizes.

## Discussion

During the past 2 decades, significant advancements have been made toward understanding MD. The association between the enlargement of lacrimal and salivary glands and elevated IgG4 serum levels was first reported in 2004 [8] and was later confirmed by several studies globally [9,10]. MD is now considered to be an ophthalmic manifestation of IgG4-RD [11], a novel clinical entity characterized by the infiltration of IgG4-immunopositive plasmocytes and elevated serum IgG4 concentrations, accompanied by the enlargement of various organs and the formation of masses within the organs [12]. MD typically presents with peri-ocular tissue swelling and orbital myositis, as well as submandibular and/or parotid gland enlargement [13]. The disease typically manifests bilaterally and symmetrically. The most commonly involved organs are the lacrimal glands, followed by the submandibular and parotid glands. On ultrasound, multiple hypoechoic areas were observed within the glands and hypervascularity was also detected. On computed tomography scans revealed homogeneous attenuation, the enhancement of tumor-like lesions, and the enlargement of the lacrimal or salivary glands [13]. On MRI, the glands appeared as isointense compared with skeletal muscle on T1-weighted imaging and typically appeared with low to intermediate signal intensity on T2-weighted imaging due to increased cellularity and fibrosis [14]. Glands were homogenously enhanced on contrast images. Histopathological analyses reveal that despite massive inflammatory cell infiltration, almost no acinar cells or duct apoptosis were observed until tissue fibrosis became very advanced [5]. Lymphoid follicles may be numerous, in some cases. Diffuse lymphoplasmacytic and plasma cell infiltration is usually marked, and storiform fibrosis and bilateral phlebitis are histological features of IgG4-RD [15]. Immunostaining can reveal IgG-positive cells, using either IgG staining or CD138 staining, and immunohistochemical staining for IgG4 showed IgG4-positive plasmacytic infiltration [15].

The 2019 American College of Rheumatology/ European League Against Rheumatism classification criteria for IgG4-RD is recommended for diagnosis. If the inclusion criteria are met, no exclusion criteria are present, and the total score is ≥20, then a diagnosis of IgG4-RD could be established [15]. Alternatively, the diagnostic criteria for ophthalmic IgG4-RD could be applied, as follows [16]. 1) Imaging studies show the enlargement of the lacrimal gland, trigeminal nerve, or extraocular muscle, in addition to the presence of masses or hypertrophic lesions or the enlargement of various ophthalmic tissues. 2) Histopathologic examination shows marked lymphoplasmacytic infiltration, and sometimes fibrosis, including the identification of IgG4 plasmocytes, which satisfies the following criteria: the ratio of IgG4-positive cells to IgG-positive cells is 40% or greater, or more than 50 IgG4-positive cells can be counted in a high-power field (x400). 3) The blood test shows elevated serum IgG4 levels (≥135 mg/dL). Many diseases can present with the nonspecific symptoms of lacrimal and salivary gland enlargement, especially inflammatory diseases, such as Sjogren´s syndrome. Imaging studies performed in Sjogren´s syndrome are typically normal at early stages, whereas later stages are typically associated with dry eyes and mouth. On MRI, the affected gland has a “salt and pepper” or “honeycomb” appearance on T2-weighted images.

Systemic corticosteroid is the first-line treatment for MD, in most of the cases. The majority of patients respond well to glucocorticoid therapy within weeks, typically with reductions in mass sizes and improved symptoms, often associated with a significant drop in serum IgG4 levels [17]. Intraorbital glucocorticoid injections have also been reported as an alternative approach for diseases centered in the anterior orbit to avoid general immunosuppression [18]. In contrast, immunomodulatory drugs are highly effective for the treatment of IgG4-RD. However, this treatment is typically reserved as a second-line therapy due to high costs and potential side effects [18,19]. In spite of a generally good response to glucocorticoid therapy, the recurrence of ophthalmic IgG4-RD is not uncommon, with a documented relapse rate from 17% to 50% [17]. The long-term prognosis of MD remains unknown. A case of bilateral ophthalmic IgG4-RD that evolved into mucosa-associated lymphoid tissue (MALT) lymphoma was reported [20], but whether ophthalmic IgG4-RD predisposes the development of ocular IgG4 MALT lymphomas remains uncertain. Further studies are warranted to better understand the relationship between ophthalmic IgG4-RD and IgG4 MALT lymphoma. Our first patient reported that the onset of symptoms was associated with her first pregnancy, which may be associated with immunological changes during pregnancy. However, the etiology of the second patient was unknown. Both patients presented with typical symptoms and imaging characteristics consistent with MD and both demonstrated good responses to steroid therapy.

## Conclusion

MD is a benign disease of unknown etiology, characterized by the bilateral enlargement of the lacrimal and salivary glands. The symptoms could be expected to improve following steroid therapy. We presented a case report of MD with a focus on the imaging. In our article, the association between lacrimal biopsy tissues, IgG4 serum levels, and treatment response were consistent with IgG4-RD. Ultrasound imaging may be a useful procedure for monitoring the efficacy of corticosteroid therapy in these patients.

## References

[1] Rao D, Natter P, Fernandes R, Wang ZB, Sandhu SJS (2017). A Case Report of Mikulicz Syndrome. J Radiol Case Rep.

[2] Mikulicz J (1892). Beiträge zur Chirurgie: Festschrift gewidmet Theodor Billroth. Med Classics.

[3] Schaffer AJ, Jacobsen AW (1927). Mikulicz's syndrome: a report of ten cases. American Journal of Diseases of Children.

[4] Sjögren H (1938). Zur kenntnis der keratoconjunctivitis sicca IV: Mikroskopische untersuchungen über das initialstadium der drüsenveränderungen. Acta Ophthalmologica.

[5] Tsubota K, Fujita H, Tsuzaka K, Takeuchi T (2000). Mikulicz´s disease and Sjogren´s syndrome. Invest Ophthalmol Vis Sci.

[6] Yamamoto M, Takahashi H, Shinomura Y (2011). Mikulicz's disease and its extraglandular lesions. Current Immunology Reviews.

[7] Umehara H, Okazaki K, Masaki Y, Kawano M, Yamamoto M, Saeki T (2012). Comprehensive diagnostic criteria for IgG4-related disease (IgG4-RD), 2011. Mod Rheumatol.

[8] Yamamoto M, Ohara M, Suzuki C, Naishiro Y, Yamamoto H, Takahashi H (2004). Elevated IgG4 concentrations in serum of patients with Mikulicz's disease. Scand J Rheumatol.

[9] Plaza JA, Garrity JA, Dogan A, Ananthamurthy A, Witzig TE, Salomão DR (2011). Orbital inflammation with IgG4-positive plasma cells: manifestation of IgG4 systemic disease. Arch Ophthalmol.

[10] Deschamps R, Deschamps L, Depaz R, Coffin-Pichonnet S, Belange G, Jacomet PV (2013). High prevalence of IgG4-related lymphoplasmacytic infiltrative disorder in 25 patients with orbital inflammation: a retrospective case series. Br J Ophthalmol.

[11] Umehara H, Okazaki K, Masaki Y, Kawano M, Yamamoto M, Saeki T (2012). A novel clinical entity, IgG4-related disease (IgG4RD): general concept and details. Mod Rheumatol.

[12] Kubo K, Yamamoto K (2016). IgG4-related disease. Int J Rheum Dis.

[13] Stone JH, Zen Y, Deshpande V (2012). IgG4-related disease. N Engl J Med.

[14] Fujita A, Sakai O, Chapman MN, Sugimoto H (2012). IgG4-related disease of the head and neck: CT and MR imaging manifestations. Radiographics.

[15] Wallace ZS, Naden RP, Chari S, Choi H, Della-Torre E, Dicaire JF (2020). The 2019 American College of Rheumatology/European League Against Rheumatism Classification Criteria for IgG4-Related Disease. Arthritis Rheumatol.

[16] Goto H (2015). Takahira M, Azumi A, Japanese Study Group for IgG4-Related Ophthalmic Disease. Diagnostic criteria for IgG4-related ophthalmic disease. Jpn J Ophthalmol.

[17] Yu WK, Tsai CC, Kao SC, Liu CJ (2018). Immunoglobulin G4-related ophthalmic disease. Taiwan J Ophthalmol.

[18] Andrew NH, Gajdatsy A, Selva D (2016). Intraorbital corticosteroid injection for the treatment of IgG4-related ophthalmic disease. Br J Ophthalmol.

[19] Lang D, Zwerina J, Pieringer H (2016). IgG4-related disease: current challenges and future prospects. Ther Clin Risk Manag.

[20] Mulay K, Aggarwal E (2014). IgG4-related dacryoadenitis evolving into an extra-nodal, marginal zone B-cell lymphoma (EMZL): a tale of two lacrimal glands. Pathology.

